# Therapy Development for Spinal Muscular Atrophy in SMN Independent Targets

**DOI:** 10.1155/2012/456478

**Published:** 2012-05-31

**Authors:** Li-Kai Tsai

**Affiliations:** ^1^Department of Neurology, National Taiwan University Hospital and National Taiwan University College of Medicine, Taipei 10002, Taiwan; ^2^Department of Neurology, National Taiwan University Hospital, Yun-lin Branch, Yun-lin 64041, Taiwan

## Abstract

Spinal muscular atrophy (SMA) is an autosomal recessive neurodegenerative disorder, leading to progressive muscle weakness, atrophy, and sometimes premature death. SMA is caused by mutation or deletion of the *survival motor neuron*-1 (*SMN1*) gene. An effective treatment does not presently exist. Since the severity of the SMA phenotype is inversely correlated with expression levels of SMN, the *SMN*-encoded protein, SMN is the most important therapeutic target for development of an effective treatment for SMA. In recent years, numerous SMN independent targets and therapeutic strategies have been demonstrated to have potential roles in SMA treatment. For example, some neurotrophic, antiapoptotic, and myotrophic factors are able to promote survival of motor neurons or improve muscle strength shown in SMA mouse models or clinical trials. Plastin-3, cpg15, and a Rho-kinase inhibitor regulate axonal dynamics and might reduce the influences of SMN depletion in disarrangement of neuromuscular junction. Stem cell transplantation in SMA model mice resulted in improvement of motor behaviors and extension of survival, likely from trophic support. Although most therapies are still under investigation, these nonclassical treatments might provide an adjunctive method for future SMA therapy.

## 1. Introduction

Spinal muscular atrophy (SMA) is characterized by motor neuron degeneration with muscular atrophy, paralysis, and an attenuated lifespan [1]. The disease is the leading genetic cause of infantile mortality [2]. SMA exhibits an autosomal recessive pattern of inheritance with an incidence of 1 in 6,000–10,000 newborns and a carrier frequency of about 1 : 35 [2, 3]. Based on age of onset and achievement of motor milestones, SMA has been subdivided into four clinical types: severe (type I; Werdnig-Hoffmann disease), intermediate (type II), mild (type III; Kugelberg-Welander disease), and adult forms [4]. Most SMA patients harbor deletions, mutations, or conversions of the telomeric copy of the *survival motor neuron* gene (*SMN1*) [5, 6]. The centromeric *SMN* gene (*SMN2*) is present in all SMA patients, but is unable to compensate for the *SMN1* gene defect as the primary transcript of *SMN2 *gene is defectively spliced [5, 6]. Currently, there are no curative therapies for SMA. Since there is an inverted correlation between the amount of SMN protein and disease severity [7, 8], SMN has been the most important therapeutic target for development of SMA treatment [9, 10]. However, some SMN independent targets and therapeutic strategies have been demonstrated to have the potential to benefit SMA [11–20]. Although most are still under investigation, these nonclassical therapies might provide an adjunctive method for future SMA therapy.

## 2. Disease Mechanisms

**Figure 1:**
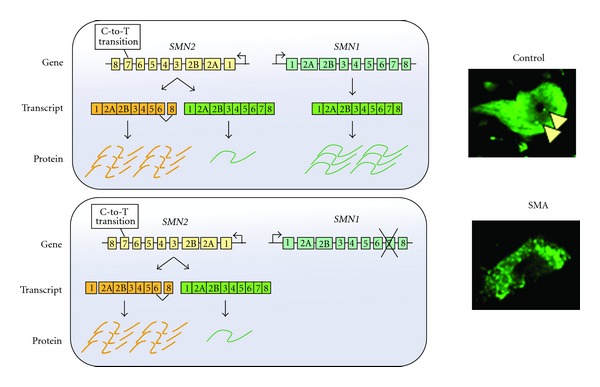
Schematic diagram of the *SMN1 *and *SMN2* genes. Humans are the only species that carry both *SMN1 *and *SMN2 *genes, located in the human 5q11.2–13.3 region [5, 168]. The *SMN1* and *SMN2* genes differ by five nucleotide exchanges [6]. Among them, a translationally silent cytosine to thymidine exchange at position 6 of exon 7 is responsible for the skipping of exon 7 during splicing of the* SMN2* gene [6]. The C-to-T transition abolishes an exonic splice enhancer site and generates a new exonic splicing silencer domain for the last coding exon [169, 170]. Subsequently, through alternative splicing, most of the translating SMN protein from the *SMN2* gene lacks the C-terminal residue and becomes less stable and relatively inactive [171]. In normal situation, abundant SMN protein is produced mainly from *SMN1* gene with a little amount from *SMN2* gene. The spinal motor neuron from a wild-type mouse thus expresses a high level of SMN in both cytoplasm and nucleus with several gems (arrow head) as compared to that in an SMA mouse. With homozygous mutation of the *SMN1 *genes, all SMA patients still have at least one *SMN2* gene copy [6]. While complete loss of SMN expression is embryonically lethal [172], the small amount of full-length SMN protein produced by the *SMN2* gene (about 20%) prevents lethality in SMA patients, but has insufficient SMN levels to assist in recovery from spinal motor neuron death [28].

Although pathogenesis of SMA has been investigated extensively, some of the detailed disease mechanisms are still not fully understood.  Figure 1 showed the genetics in SMA. The SMN is a 38-kDa protein expressed in both the cytoplasm and nucleus of all cells [21]. SMN serves as a chaperone in the assembly of spliceosome precursors by combining small nuclear RNA (snRNA) molecules with Sm proteins to generate small nuclear ribonucleoproteins (snRNPs) [22, 23]. The snRNP assembly activity is dramatically reduced in spinal cord from SMA model mice and the degree of snRNP assembly impairment correlates with disease severity [24]. Therefore, SMN plays a critical role in pre-mRNA splicing. Evidence shows that SMN is also involved in the stabilization and maturation of the neuromuscular junction and the transportation of axonal mRNAs in motor neurons [25–27]. SMN-deficient motor neurons exhibit severe defects in clustering voltage-gated calcium channels in axonal growth cones [26]. An alteration of calcium channel distribution might influence neurotransmitter release, causing dysfunction and immaturation of neuromuscular junction [25, 28]. In addition, the SMN protein can form granules that are transported and associated with *β*-actin mRNA in neuronal processes [29]. The close relationship of SMN and *β*-actin has further demonstrated that motor neurons derived from SMA model mice have shortened axons and small growth cones, which are also deficient in *β*-actin mRNA and protein [30]. Therefore, SMN has a function in maintaining proper neuronal machinery via assistance in splicing process and establishing adequate communication between the muscles and nerves at the motor end plate through stabilization of the neuromuscular junction. The loss of maintenance and communication might thus trigger the cascade of events that probably results in motor neuron death.

SMA mouse models have been generated through mouse *Smn* knockout and human *SMN2 *transgenic methods [8, 31]. These mice reveal spinal motor neuron degeneration, muscle atrophy, and impaired motor performances similar to SMA patients. The disease severity of these SMA mice is also inversely correlated to the copy number of the *SMN2* transgenes [8, 31]. These findings confirm that SMA is directly caused by SMN deficiency. Denervation of neuromuscular junction precedes spinal motor neuron loss in SMA mice [25]. Neuromuscular junction can form and function normally prior to the postnatal onset of disease [32]. Afterward, abnormal neurofilament accumulation and functional disruption at the neuromuscular junction become evident [25]. Alongside these morphological and functional changes at the neuromuscular junction, studies on the spinal cord of SMA mice showed an apparent failure of expression of genes that cluster in postnatal developmental pathways [33]. Subsequently, through still unknown mechanisms, motor neurons degenerate in spinal anterior horn regions probably through cell apoptosis [16, 34], and muscle atrophy and motor dysfunction become apparent.

Recently, congenital heart defects have been recognized as additional important phenotypes especially in type I SMA patients, including atrial septal defects, dilated right ventricle, and ventricular septal defects [35]. The histological studies in SMA model mice also showed that cardiac remodeling starts at the embryonic stage in the severe SMA mice while motor neurons are not yet visibly affected at this stage. After birth, there is progressive cardiac fibrosis, which may result from oxidative stress [36]. SMA mice also suffer from severe bradyarrhythmia characterized by progressive heart block and impaired ventricular depolarization, which may be related to defective sympathetic innervation [37]. Notably, systemic restoration of SMN expression is able to diminish the cardiac defects accompanied with prolonged lifespan, implying that cardiac abnormalities are playing a critical role on SMA pathogenesis [38, 39].

## 3. SMN Dependent Therapy

Since SMN levels generally correlate with disease severity in SMA patients and mouse models [7, 8, 31, 40], SMN is the best therapeutic target for development of SMA treatment. Various strategies to increase the SMN levels have been tested in SMA mouse models and some of them have even showed promising beneficial effects [9, 10]. Until now, none of them have been demonstrated to be consistently robust or produce continual benefits in SMA patients. These therapeutic strategies are divided into small molecules, antisense oligonucleotides (ASO), and viral vector-mediated gene therapy.

All SMA patients have at least one copy of the *SMN2* gene, providing an opportunity for manipulation of the *SMN2 *gene expression [6]. The mode-of-action for a potential SMA therapy using small molecules mainly includes restoration of the *SMN2* splicing pattern, activating the *SMN2* promoter, and extending the half-life of SMN mRNA or protein [10]. The potential drugs include histone deacetylase (HDAC) inhibitors such as sodium butyrate [41], phenylbutyrate [42], valproic acid (VPA) [43], trichostatin A [44], SAHA [45], and LBH589 [46], as well as hydroxyuria [47], sodium vanadate [48], aclarubicin [49], indoprofen [50], bortezomib [51], and aminoglycosides, such as tobramycin, amikacin [52], TC007 [53], and G418 [54]. Since there are still no drugs that have shown consistent benefits in clinical trials [55, 56], finding an effective treatment with distinct therapeutic mechanisms, such as SMN independent targets, is necessary for future SMA therapy.

Among these small molecules, VPA is the drug being studied most extensively and has been used in patients with epilepsy and bipolar disorders for decades [57]. VPA treatment increased levels of SMN transcripts and protein in fibroblasts derived from SMA patients through upregulation of serine/arginine-rich (SR) proteins, which are involved in regulating *SMN2* exon 7 recruitment [58, 59]. Autophagy, the degradation of cytosolic components in lysosomes, maintains neuronal homeostasis; its dysfunction has been linked to various neurodegenerative diseases, possibly including SMA [60]. VPA is also an autophagy enhancer, which activated autophagic pathways and attenuated rotenone-induced toxicity in SH-SY5Y cells [61]. In addition, VPA upregulates some antiapoptotic factors such as Bcl-2 and Bcl-xl, perhaps via activation of ERK44/42 [43, 62, 63]. Probably through multiple therapeutic effects, VPA reduced motor neuron degeneration, muscle atrophy, and motor dysfunction in SMA mice [43, 64], and a small group of SMA patients showed obvious improvement in muscle strength after daily VPA treatment [65, 66]. Despite these encouraging results, large clinical trials did not confirm the beneficial effects of VPA in SMA patients [67–69]. These disappointing outcomes may contribute to different pharmacokinetics and bioavailability between rodents and humans as well as dose-limiting intolerance and drug adverse effects [9]. In addition, the responses of VPA treatment showed intrapatient and interpatient variability in the study using fibroblasts and lymphoblasts from SMA patients [70], probably indicating that tissue and individual factors may affect the VPA effects with unknown reasons.

Using ASO to inhibit the splicing silencer for *SMN2* exon 7 leads to restoration of the normal *SMN2* splicing pattern [71]. The effects of ASO were further improved through the incorporation of a binding platform with ASO for recruitment of SR protein to the *SMN2* exon 7 region [71]. These bifunctional ASOs were able to achieve nearly 100% exon 7 inclusion and enhance SMN expression up to 2- to 3-fold in cell-based assays [72]. Injection of ASO into cerebroventricles elicited a robust induction of SMN protein in the brain and throughout the spinal cord and extended the lifespan of SMA mice [73]. A recent study demonstrated that systemic delivery of ASO resulted in dramatic prolongation of lifespan in SMA mice and the effects were much better than those with intracerebroventricular delivery of ASO (median survival, 108 versus 16 days) [39]. These findings suggest that ASO therapy has great potential in this field and extra-CNS targeting is required to rescue the SMA phenotype. However, another similar study showed different results that early intracerebroventricular delivery of ASO had a better outcome than intravenous ASO delivery [74], which suggests that therapeutic methods for ASO treatment still need further investigation and optimization.

Direct injection of adeno-associated viral vector serotype 8 (AAV8) carrying *SMN* into both cerebroventricles and upper lumbar spinal cord of SMA mice showed a robust increase in lifespan by 880% with less motor neuron degeneration and abnormal architectures of neuromuscular junction [75]. However, augmented SMN is expressed in thoracolumbar regions, but sparse in the cervical cord, which may suggest poor diffusion of AAV in subarachnoid space. In contrast, intravenous AAV serotype 9 (AAV9) injection has shown success in affecting widespread gene delivery in entire spinal cord [76]. Intravenous injection of AAV9 carrying human codon-optimized *SMN1* at postnatal day 1 recovered most motor function, neuromuscular physiology, and lifespan in SMA mice [77]. Notably, postnatal day 1 treatment resulted in the maximal transduction of the motor neurons, while postnatal day 10 treatment led to glia-predominant transduction [77]. This shift in cell type specificity was probably because of the closure of the blood brain barrier that occurs within the first week of life in neonatal mice [78]. When the blood brain barrier is mature and patent, virions are probably not able to penetrate out of vessels smoothly to access motor neurons, but only encounter the endothelial wrappings of astrocyte end feet. Since blood brain barrier likely matures in as early as human neonatal period [79], the AAV9 transduction efficacy should further be tested in nonhuman primates of different ages to identify the optimal temporal window for future therapy.

## 4. SMN Independent Targets and Treatment

### 4.1. Neuroprotection, Antiapoptosis, and Myotrophic Effects

#### 4.1.1. Insulin-Like Growth Factor-1

Insulin-like growth factor-1 (IGF-1) is a trophic factor mainly secreted by the liver and circulates at high levels in the bloodstream. IGF-1 is a key molecule involved in normal brain growth and function [80] and may have a neuroprotective effect by inhibiting neuronal death in Huntington's disease and spinocerebellar ataxia [81, 82]. IGF-1 also enhances axonal outgrowth of corticospinal motor neurons [83]. *Igf1*-null mice show some phenotypic similarity to SMA mice, such as small size and generalized muscle dystrophy, with most of them dying at birth [84]. Notably, serum IGF-1 level was decreased in SMA mice, and systemic increase of SMN expression using the ASO strategy in SMA mice was accompanied with restoration of serum IGF-1 to normal levels [39]. Interestingly, mRNA levels of IGF-binding protein, acid labile subunit (IGFALS), but not IGF-1, was reduced in SMA mice. IGFALS binds to IGF-1 and IGF-binding protein 3 to form a stable ternary complex, extending the half-life of IGF-1 from 10 minutes to 12 hours [85]. Therefore, the low serum IGF-1 level in SMA mice is likely related to downregulation of IGFALS, and IGF-1 may be one of the factors that contribute to the pathogenesis of SMA [39].

IGF-1 treatment has been shown to improve disease phenotypes in rodent models of motor neuron diseases such as amyotrophic lateral sclerosis (ALS) [86] and spinal and bulbar muscular atrophy (SBMA) [87]. For SMA, transgenic expression of IGF-1 in skeletal muscle of SMA mice resulted in an increase in myofiber size and a modest improvement in median survival [11]. Delivery of a plasmid DNA vector encoding IGF-1 by intracerebroventricular injection into newborn SMA mice also increased body mass and provided a modest improvement in median survival [12]. However, intracerebellar viral delivery of IGF-1 reduced motor neuron degeneration, but did not improve motor function in the mildly affected SMA mice [88]. Therefore, the effects of IGF-1 and IGFALS-related therapy using different treatment strategies in SMA still require further investigation.

#### 4.1.2. Ciliary Neurotrophic Factor

Schwann cells close to neuromuscular endplates play a major role in triggering terminal sprouting [89]. These cells express ciliary neurotrophic factor (CNTF), and lack of CNTF expression strongly reduces terminal sprouting and motor unit size [13]. In a mouse model of ALS, the depletion of synaptic vesicles precedes the loss of synapses; CNTF could prevent the depletion of synaptic vesicles and thus maintain function of neuromuscular junctions [90]. CNTF treatment using CNTF-secreting stem cells or by local CNTF injection into skeletal muscle led to better maintenance of peripheral motor axons in a mouse mutant, progressive motor neuronopathy (*pmn*) [91, 92].

In a severe type of SMA mice, the sprouting and enlargement of motor units do not normally occur. In contrast, the architecture and function of neuromuscular junctions in heterozygous* Smn* (+/−) mice are relatively preserved, despite some loss of spinal motor neurons [13]. However, completed knockout of CNTF in heterozygous *Smn* (+/−) mice reduces the sprouting responses of the nerve terminals accompanied with reduced muscle strength [13]. These results imply that CNTF may be able to compensate loss of motor neurons by sprouting from remaining motor axon terminals so that neuromuscular endplates remain innervated; CNTF may thus guide the way for new therapies for SMA. Although systemic CNTF treatment elicited severe adverse effects including fever and cachexia in ALS patients [93], muscle or CNS targeting CNTF therapy might offer a chance to reduce these side effects and show benefits in SMA.

#### 4.1.3. Cardiotrophin-1

CNTF and Cardiotrophin-1 (CT-1) are both members of the IL-6 family, which bind a common receptor complex requiring leukemia inhibitory factor receptor (LIFR) and gp130 [94]. CT-1, an important cardioprotective cytokine, also has beneficial effects in neuromuscular systems [95]. CT-1 is essential in normal motor neuron development and is also able to support long-term survival of motor neurons as demonstrated in culture cells and rats with axotomy [96]. In addition, overexpression of CT-1 in *pmn* and ALS mice both significantly delayed disease onset, reduced degeneration of motor neurons and axons, and preserved the terminal innervation of skeletal muscles [97, 98]. For SMA mice, intramuscular injection of adenoviral vector expressing CT-1, even at very low doses, prolonged survival, delayed motor defects, and diminished motor axonal degeneration and aberrant synaptic terminals [14]. Although most of studies regarding CT-1 are focused on diseases in the cardiovascular system, CT-1 might still be a valuable therapeutic agent for motor neuron diseases through neurotrophic effects.

#### 4.1.4. Bcl-xL and Bax

Degeneration of spinal motor neurons in SMA is mediated in part through apoptosis [16, 34]. In the Bcl-2 family, Bcl-xL and Bax are important regulators of cell death in the nervous system when cells have matured. Bcl-xL is an antiapoptotic member of the Bcl-2 family and acts by inhibiting proapoptotic members of the Bcl-2 family through heterodimerization [99]. Bcl-xL was downregulated in SMA patients and model mice [17, 100]. Bcl-xL overexpression can protect against motor neuron death in cultured primary motor neurons [101] and embryonic motor neurons with *SMN *knockdown [102]. Interestingly, Bcl-xL overexpression in SMA mice reduced motor neuron degeneration, preserved motor function, and prolonged lifespan without changes in SMN expression levels [17]. In addition, Bax protein is a major proapoptotic member of the Bcl-2 family. *Bax* knockout SMA mice had milder disease severity and longer lifespan with less spinal neuronal degeneration than SMA littermates with wild-type *Bax* genes [16]. Therefore, effects of Bcl-xL and Bax may not be simply through apoptotic pathways, but through unknown mechanisms to salvage neural function in SMA. The ratio of Bcl-xL/Bax is thus another attractive target, where the potential to increase Bcl-xL and decrease Bax expression may be of benefit to SMA patients.

#### 4.1.5. Riluzole

Riluzole, a 2-aminobenzothiazole, is the only disease-modifying therapy available for ALS [103]. Although riluzole is known to modulate excitatory neurotransmission mainly through inhibition of glutamate release, the precise neuroprotective mechanisms remain largely speculative [104]. In SMA mice, riluzole improved median survival and reduced aberrant cytoskeletal organization of motor synaptic terminals [105]. However, a small phase I clinical trial, enrolling 7 riluzole-treated and 3 placebo-treated type I SMA infants, demonstrated no significant differences in survival and the change in motor abilities after riluzole treatment [106]. Nevertheless, further analysis showed that 3 patients in the riluzole group presented an unusual disease course and were still alive at the age of 30 to 64 months. The pharmacokinetics of riluzole in SMA patients has recently been investigated [107], and the long-term benefits of riluzole still warrant large clinical trials for SMA patients.

#### 4.1.6. Gabapentin

Gabapentin is a GABA analogue and has been used clinically for patients with seizures and neuropathic pain for more than 10 years [108]. Gabapentin could also have a neuroprotective action in part by reducing the pool of releasable glutamate in neurons, thereby diminishing the excitotoxicity potential [109, 110]. Although gabapentin treatment showed marginal reduction in disease progression in a phase II clinical trial for ALS patients [111], the following phase III clinical trial did not reveal significant benefits after gabapentin treatment for 9 months [112]. For SMA, the first clinical trial of gabapentin enrolled 84 type II and III SMA patients. There was no difference between the gabapentin and placebo groups in any outcome measure including changes in muscle strength, pulmonary function, or motor functional rating scale after 12 months of treatment [113]. However, another clinical trial which enrolled 120 type II and III SMA patients showed a significant improvement in muscle strength of legs at both 6 and 12 months after gabapentin treatment [114]. Meta-analysis of these two trials did not successfully demonstrate the beneficial effects of gabapentin in SMA [56].

#### 4.1.7. *β*-Adrenergic Agonist

*β*2-Adrenergic agonist, such as salbutamol (albuterol in the United States), enhanced muscle strength in aged rats [115], human healthy volunteers [116], and some pathological conditions [117, 118]. In a pilot clinical trial, thirteen type II or III SMA patients receiving salbutamol for 6 months showed significant increase in myometry, forced vital capacity, and lean body mass [119]. A further larger trial enrolling 23 type II SMA patients consistently got similar results that functional scores were better after daily salbutamol treatment for 6 or 12 months [120]. Notably, the drug did not produce any major side effects [119, 120]. The mechanism of action of *β*2-adrenergic agonists on human skeletal muscles to enhance muscle strength is not completely understood. Interestingly, salbutamol also promoted exon 7 inclusion in *SMN2* transcripts and thus increased levels of full-length transcripts of *SMN2* in SMA fibroblasts [121]. In SMA patients, daily salbutamol significantly and consistently increased *SMN2* full-length transcript levels in peripheral leukocytes, and the response was directly proportional to *SMN2 *gene copy number [122]. Considering bifunctional therapeutic effects and safety of salbutamol, large randomized double-blinded placebo-controlled clinical trials are mandatory.

#### 4.1.8. Follistatin

Myostatin is a member of the TGF-*β* family and functions as a potent negative regulator of muscle growth [123]. Inhibition of myostatin increases muscle mass and strength in wild-type rodents and improves the pathophysiology of a mouse model for muscular dystrophy [124, 125]. Follistatin is a cystine-rich glycoprotein, which binds to and inhibits several TGF-*β* family members, including myostatin [126]. Follistatin delivered by intramuscular injection of recombinant viral vectors increased muscle mass in mouse models of both ALS and Duchenne muscular dystrophy [127, 128]. Since SMA also features diffuse muscle atrophy, inhibition of myostatin may also be a therapeutic strategy. Intraperitoneal injection of recombinant follistatin in SMA model mice increased muscle mass, improved motor function, and prolonged lifespan by 30% without changes in SMN protein levels in spinal cord and muscles [15]. However, other studies detected no phenotypic alteration in transgenic overexpression of follistatin or ablation of myostatin in SMA mice [129, 130]. The reason for this discrepancy is unclear and the effects of follistatin for SMA treatment still need further validation.

### 4.2. Axonal Dynamics

#### 4.2.1. Plastin-3

Although SMA-affected siblings usually develop similar disease severity in terms of their age at onset and the progression of disease [131], a small proportion of individuals with homozygous *SMN1 *mutation are fully asymptomatic despite carrying an identical number of *SMN2* copies as their affected siblings, suggesting the influence of modifier genes [132, 133]. The first potential SMN-independent disease modifier, plastin-3, was recently identified from six SMA-discordant families with eight fully asymptomatic females who had inherited the same *SMN1* and *SMN2* alleles as their affected siblings [18]. Increased levels of plastin-3 were also found to correlate with a mild SMA phenotype in female patients, independently of SMN protein levels [18, 134].

 Plastin-3, an actin binding protein, is a regulator of actin filament organization and is expressed in almost all solid tissues, including the human brain, spinal cord, and muscles [18]. Plastin-3 colocalizes with SMN in granules throughout motor neuron axons, and plastin-3 protein levels are reduced in brain and spinal cord of an SMA mouse model [18, 135]. In SMN-depleted neuronal PC12 cells and primary mouse motor neuron cultures derived from SMA mice, plastin-3 overexpression was able to recover from axon outgrowth defects [18]. Notably, overexpression of plastin-3 or its orthologues also led to diminishment of axon defects and disease severity in SMN depleted zebrafish embryos, *Drosophila*, and *C. elegans* [18, 136]. SMN has been shown to moderate and restrict the negative function of profilin IIa on actin polymerization [137]. Profilin IIa is another actin binding protein, and knockdown of profilin IIa results in stimulation of neurite outgrowth, while overexpression of profilin IIa reduces neurite number and size [138]. Knockout of profilin IIa in SMA model mice was able to restore abnormal low plastin-3 levels. However, the phenotype of these SMA mice was not ameliorated despite the depletion of profilin IIa and restoration of plastin-3 levels, which suggests that other components of actin dynamics are also critically affected in SMA [135]. Although some questions need to be answered, such as the mechanisms behind plastin-3 in SMA and effects of plastin-3 upregulation in SMA mouse models, plastin-3 may become an important SMN-independent therapeutic target for SMA in the future.

#### 4.2.2. Cpg15

The candidate plasticity-related gene 15 (cpg15) is highly expressed in the developing ventral spinal cord and can promote motor axon branching and neuromuscular synapse formation [139, 140]. Cpg15 mRNA colocalizes with SMN protein in axons and is locally translated in growth cones [141]. HuD is a neuron-specific RNA-binding protein and also an interacting partner of SMN [141–143]. Cpg15 may be an mRNA target for the SMN-HuD complex and SMN deficiency reduced *cpg15* mRNA levels in neurons [141]. Most importantly, cpg15 overexpression partially recovered from motor axonal deficits in zebrafish with SMN deficiency [141]. Therefore, cpg15 appears to be a crucial downstream effecter of SMN in neurons and may serve as a modifier of SMA disease by regulating axon extension and axon terminal differentiation.

#### 4.2.3. Rho-Kinase Inhibitor

Rho-kinase signaling is a major regulatory pathway of actin dynamics, and Rho-kinase activation is associated with dendritic simplification, and reduced spine length and density [144]. Rho-kinase activity is upregulated in SMN-depleted PC12 cells and SMA model mice [145, 146]. The migratory capacity of the U87MG astroglioma cells was attenuated by knockdown of SMN through abnormal activation of Rho-kinase pathway [147]. Normally, SMN binds to profilin IIa to form complexes, and Rho-kinase may phosphorylate profilin IIa [148]. Through competition between SMN and Rho-kinase for binding to profilin IIa, SMN deficiency results in a decrease in SMN-profilin IIa complexes and stronger interaction of profilin IIa with Rho-kinase [148]. Subsequently, hyperphosphorylation of profilin IIa in SMA leads to inhibition of neurite outgrowth. Therefore, Rho-kinase inhibition might be able to correct the effect of SMN reduction in SMA to achieve an adequate ratio of de-/phosphorylated profilin IIa.

 Notably, treatment of SMA model mice with Rho-kinase inhibitor Y-27632 or Fasudil led to a significant prolongation in survival, improvement in integrity of neuromuscular junction, and increase in muscle fiber size without altered SMN expression or increase in the number of spinal motor neurons [146, 149]. Since Fasudil has been successfully applied in many clinical trials for other neurological and vascular diseases based on its neuroprotection, vasodilatation, and immune modulation effects [150], the results of Fasudil therapeutic studies for SMA patients are anticipated.

### 4.3. Stem Cells

#### 4.3.1. Neural Stem Cells

A diagnosis of SMA is usually made following a patient's initial presentation of muscle weakness, at which there would be substantial spinal motor neuron loss [64]. Both SMN dependent and independent treatments described above could only prevent disease progression, but not regain lost motor neurons, while stem cell therapy might provide a possibility for cell replacement. Fetal-derived neural stem cells (NSCs) are able to self-renew and are multipotent with the capacity of producing neurons (including motor neurons), astrocytes, and oligodendrocytes [151]. NSCs can be isolated from mouse embryonic spinal cords and differentiated toward a motor neuron cell fate by priming with retinoic acid and sonic hedgehog. Intrathecal injection of these primed NSCs in *nmd *mice, another model of motor neuron disease, resulted in improvement of abnormal phenotypes and extension of survival [152]. In addition, NSCs derived from human fetal spinal cord delayed disease onset and prolonged lifespan after being transplanted directly into spinal cord of ALS mice [153, 154].

 In a severe type of SMA mouse model, intrathecal injection at postnatal day 1 with primed NSCs derived from mouse embryonic spinal cord also promoted motor neuron survival, improved motor function, and prolonged lifespan [19]. Although some grafted cells expressed motor neuron markers, there was no direct evidence suggesting that the beneficial effects resulting from the formation of functional motor units by the transplanted cells. Transplantation of undifferentiating NSCs also showed a significant increase in survival of SMA mice, although not as efficient as the effects of NSCs primed into a motor neuron fate [19]. Therefore, the observed benefits of NSCs in SMA model mice were likely related to trophic support.

#### 4.3.2. Embryonic Stem Cells

Although fetal-derived NSC transplantation in SMA mice showed promising effects, their derivation from a spinal cord source impedes further clinical implementation because of ethical and technical issues [155]. On the other hand, embryonic stem cells might be easier to obtain and are also able to differentiate *in vitro* and *in vivo* into NSCs and a motor neuron fate [156]. Intraspinal grafting of embryonic stem cell-derived motor neurons resulted in a significant improvement in motor behaviors in the ALS rat [157]. For SMA, embryonic stem cell-derived NSCs transplanted intrathecally in SMA model mice migrated to spinal anterior horn and improved motor function and lifespan [20]. Although the grafted stem cells integrated appropriately into the parenchyma, and expressed both neuron- and motor neuron-specific markers, there was again no evidence of newly generated motor neuron outgrowth to the muscles. In one previous study, a boy with ataxia telangiectasia received intracerebellar and intrathecal injection of human fetal NSCs. Four years later, he was diagnosed with a donor-derived multifocal brain glioneuronal neoplasm [158]. To increase the differentiation rate of embryonic stem cells into NSCs before transplantation, the above SMA study used drug-selectable embryonic stem cell lines that ganciclovir and G418 have been applied for selection against undifferentiated embryonic stem cells and for neuroepithelial cells, respectively. Usage of these drug-selectable stem cells not only promoted transplantation safety, but also produced superior treatment results as compared to using wild-type embryonic stem cells [20].

#### 4.3.3. Induced Pluripotent Stem Cells

Since the first report on reprogramming of mouse fibroblasts into so-called induced pluripotent stem (iPS) cells by the expression of oct3/4, Sox2, c-Myc, and Klf4 in 2006 [159], reprogramming of human somatic cells to a pluripotent state was achieved using similar approaches [160, 161]. The iPS cells can be differentiated into cells of endodermal, mesodermal, or ectodermal origin, and further lineage restriction can obtain specific neural subtypes or astrocytes. Recently, iPS cells have been successfully generated from fibroblasts of SMA patients [162, 163]. The SMA-specific iPS cells exhibited a reduced capacity to form motor neurons and an abnormality in neurite outgrowth that ectopic SMN expression rescued these abnormal phenotypes [163]. These iPS cells provide a novel opportunity in disease modeling for investigating SMA pathogenesis and can be used in screening novel compounds for SMA treatment.

 The use of fetal-derived cells or embryonic stem cells for transplantation is hurdled by problems of availability, the possibility of immune rejection, and ethics. In contrast, the source of iPS cells is unlimited, and iPS cells can be transplanted autologously. Transplantation of normal neurons derived from iPS cells reduced abnormal phenotypes in a murine model of Parkinson's disease [164]. Notably, when iPS cell-derived neural precursor cells from a patient with Parkinson's disease were transplanted into the striatum of a Parkinson's disease rat model, the donor cells differentiated into dopaminergic neurons, survived in the rodent brain for several months, and reduced the abnormal motor asymmetry [165]. For autologous iPS cell transplantation in SMA, iPS-derived neural precursor cells or motor neurons should be pretreated to express a high level of SMN before transplantation. Until now, there is still no cell transplantation report using iPS cells in SMA.

## 5. Conclusions

In various neurological disorders, many diseases, such as Parkinson's disease, epilepsy, and multiple sclerosis, are treated clinically with multiple drugs in combination to enhance the therapeutic effects. Motor neurons may also require additional support to optimally respond to SMN-based treatment. In the past two decades, there has been tremendous progress in SMA regarding genetics, pathophysiology, and therapeutics. Some useful strategies to enhance SMN expression have been developed, and some novel SMN-independent therapeutic targets have been discovered. While SMN acts to modulate and correct the neuromuscular junction for functional improvement, SMN-independent targets could play a role of extension in the survival of motor neurons and reduce the influence of SMN depletion in axonal dynamics.

The two currently available stem cell transplantation studies for SMA have only demonstrated benefits likely with trophic support without evidence of functional cell replacement [19, 20]. To generate functional motor units, the grafted stem cells should be able to differentiate into motor neurons, appropriately project the axons a long distance toward corresponding muscles, and form functional synapses within neuromuscular junctions. In a virus-induced rat model of motor neuron degeneration, mouse embryonic stem cell-derived motor neurons transplanted into spinal cord could survive, extend axons, form functional motor units, and promote recovery from paralysis [166, 167]. The successful development of motor units in the above studies may result from a combination approach, which includes administration of dibutyryl-cAMP, rolipram, cyclosporine, and glial cell line-derived neurotrophic factors to promote motor neuron survival, circumvent myelin repulsion, prevent immune rejection, and enhance axonal outgrowth, respectively. Therefore, cell replacement therapy using stem cells for SMA is not totally impossible; however, there is still much to be accomplished in cell therapy before being applied clinically to treat motor neuron diseases.
